# Healthspan Improvements in *Caenorhabditis elegans* with Traditional Chinese Herbal Tea

**DOI:** 10.1155/2020/4057841

**Published:** 2020-12-16

**Authors:** Chunxiu Lin, Xiaoying Zhang, Chuting Zhuang, Yugui Lin, Yong Cao, Yunjiao Chen

**Affiliations:** ^1^Guangdong Provincial Key Laboratory of Nutraceuticals and Functional Foods, College of Food Science, South China Agricultural University, Guangzhou, Guangdong, China; ^2^Guangdong Laboratory for Lingnan Modern Agriculture, Guangzhou, 510642 Guangdong, China; ^3^Department of Microbiology, Guangxi Medical University, Nanning, China

## Abstract

Searching for natural and safe herbal tea with health benefits has attracted more and more attention, which is of great significance for reducing disease risk. A Chinese traditional herbal tea (HT) is rich in active ingredients extracted from natural plants. Numerous pharmacological studies showed that HT had the potential to improve health, including antidepression and antioxidant effects. In this study, we proposed a strategy to explore the role and underlying mechanism of HT in improving healthspan of a *Caenorhabditis elegans* model. First, we found that HT significantly prolonged the lifespan without reducing fertility in worms. Second, stress resistance (oxidative stress and heat stress) was enhanced and A*β*- and polyQ-induced toxicity was relieved significantly by HT treatment. Both fat deposition and age pigment accumulation were found to be significantly reduced in HT-treated worms. The locomotion in mid-late stages was improved, indicating that behavioral mobility was also significantly enhanced. Furthermore, the main components of HT were eighteen polyphenols and two terpenoids. Finally, it was found that this protective mechanism was positively correlated with the insulin/insulin-like growth factor signaling- (IIS-) dependent manner, which went through promoting the nuclear localization of DAF-16 and its downstream SOD-3 expression. These results suggested that HT had an important role in improving health, which might serve as a promising healthy tea.

## 1. Introduction

Increased age is a critical risk factor for healthspan, such as progressive decline in tissue and organ function, neurodegeneration, and metabolic disorders. Antioxidants are important strategies for delaying aging and aging-related degenerative diseases [1]. Natural active substances, in particular, are found in herbs, conferring antioxidant activities, thereby providing beneficial effects on health. According to a report by the World Health Organization, 80% of the population in developing countries rely on traditional medicine for primary health care, and 85% of traditional medicines are derived from herb extracts [2]. Recently, medicinal herbs are increasingly explored by the food industry for their health-promoting benefits either as readily available for herbal teas or as sources of additives for functional foods and drinks [3]. Thus, herbal extract-based compositions have enormous potential to be developed as a reservoir of natural antioxidant additives and innovative health-promoting products.

Here, we explored the health effects of a Chinese traditional herbal tea (HT) made of natural herbs combined with modern technology. HT, founded in the Qing Dynasty (1828), is widely recognized as the first ancestor of herbal tea in China. HT has been widely used for a long history to treat “Shanghuo” (an unbalanced response of the body to cellular stress resulting in a series of disruptions of homeostasis) in traditional Chinese medicine [4]. In recent years, HT has been found to have various health benefits, including antistress, anti-inflammatory, and antioxidant effects [4–6]. Its main raw material is “three flowers, three grasses, and one leaf”, namely, *Dendranthema morifolium* (Ramat.) Tzvel., *Lonicera japonica* Thunb., *Plumeria rubra* L. cv. Acutifolia, *Glycyrrhiza uralensis Fisch*, *Prunella vulgaris* L, *Mesona chinensis* Benth., and *Microctis folium* (the ratio of each plant species in the formula is 3.5 : 3 : 5 : 1 : 3:5 : 5). These seven extracts were found to be of the most promising natural antioxidants, which could improve health [7–13]. There is convincing evidence that plant-derived natural antioxidants have important potential to relieve cellular stress and maintain homeostasis [14]. Therefore, it is an attractive question that HT with antioxidant activity could improve the healthspan or not.

To investigate the bioactive substances and pharmacological studies of HT, the efficient biological model *Caenorhabditis elegans* was applied. *C. elegans* are considered to be a good model for studying aging and screening for drugs with health-promoting effects [15]. It has a number of advantages, such as a simple structure, ease of feeding, a short life cycle, rapid reproduction, a large number of offspring, and distinct molecular signaling pathways [15]. Particularly, the aging process of *C. elegans* is quite conservative in evolution. For example, *C. elegans* exhibits similar physiological functions to humans, such as locomotion decreasing and harmful metabolites increasing with age. In terms of the mechanism of action of aging, multiple pathways which control longevity and stress resistance are conservative from *C. elegans* to humans, so the knowledge obtained in *C. elegans* is generally applicable to mammals including mankind [16, 17].

Therefore, the purpose of this study was to use *C. elegans*, one of the most valuable and attractive model organisms, to evaluate the potential improvement of HT for lifespan and healthspan and explore the underlying mechanism of action. This result will provide a scientific basis for the benefit of HT in improving health and delaying disease onset, valuable for studies of other plant-based products.

## 2. Materials and Methods

### 2.1. Culture of C. elegans

*C. elegans* strain N2-Bristol (wild-type); strains CL4176 (dvIs27 [myo-3p::A*β* (1-42)::let-851 3′UTR + rol-6(su1006)] X), CL2355 ([smg-1 ts (snb-1/A*β* (1-42)/long 3′-UTR)]), CL2122 (unc-54 vector + mtl-2::GFP), and AM140 (rmIs132 [unc-54p::Q35::YFP]); the transgenic worms CF1553 (muIs84(pAD76(sod-3::gfp))); and *Escherichia coli* OP50 (*E. coli* OP50) were obtained from the Caenorhabditis Genetics Center (CGC, University of Minnesota). TJ356 (zIs356 IV (pdaf-16-daf-16::gfp; rol-6)), VC433 (*sod-3*(gk235)X), and CF1038 (*daf-16* (mu86)I) were presented by Prof. Qinghua Zhou (Biomedical Translational Research Institute, Jinan University, Guangdong Province, China). All strains were cultivated on nematode growth medium (NGM) agar plates seeded with *E. coli* OP50 at 20°C unless otherwise stated. Worm synchronization was described in the previously established protocol [18]. During the worm breeding period, the worms were transferred to a new plate every day, and the rest of the time, the worms were transferred to a new plate every other day.

### 2.2. Sample Preparation

HT stock solution was provided by Guangzhou Wanglaoji Pharmaceutical Company Limited (Guangdong, China) and stored at 4°C (the analysis of various chemical constituents in HT was summarized in supplementary Table S1). The HT stock solution was mixed with *E. coli* OP50, the worm's food source, and dispersed on the NGM plates (the test concentrations were 10 mg/ml, 30 mg/ml, and 50 mg/ml). HT treatment was started from the egg stage, and the samples were harvested after 7-, 12-, and 17-day treatment unless otherwise stated.

### 2.3. Lifespan Assay

Lifespan assay was conducted as described before [18]. Approximately 60 individuals (strains N2, VC433, and CF1038) were treated with or without HT from the egg stage at 20°C. The dead worms were calculated and removed daily (from the first day of hatching). Nematodes that hatch embryos in the body (so-called “internal hatching”) or escape from the plates were not counted in the statistics [18]. Three replications were performed.

### 2.4. Fertility Assay

Fertility was measured as in the previous protocol [18]. After 48 hours with or without HT treatment, wild-type N2 worms were transferred to a new control or HT plate every 24 hours (one plate per two worms). The number of eggs laid per day was counted until no more eggs were laid. The sum of eggs per day was taken as the total number of nematode subalgebras. Three independent trials were conducted in 10 individuals per trial.

### 2.5. Stress Resistance Assay

#### 2.5.1. Oxidative Stress Resistance Assay

As previously described [19], treated on a medium with or without HT for ten days, the worms were transferred to oxidative stress plates with additional 10 mM paraquat. The survival was monitored every 12 hours. Three independent biological replicates were performed with a total of 180 worms.

#### 2.5.2. Heat Stress Resistance Assay

As previously described [19], wild-type N2 worms were treated with or without HT from the egg stage at 20°C for 4 days and then were transferred to a 35°C culture environment. The number of dead worms was recorded every hour. Sixty worms were used in each experiment for three repetitive tests.

### 2.6. Neuroprotective Assay against A*β*-Induced Toxicity

#### 2.6.1. A*β*-Induced Paralysis Assay

CL4176 strains were cultivated in NGM plates containing HT or vehicle (control) for 36 hours at 16°C and then transferred to 25°C to induce A*β* expression as described previously with minor modifications [20]. The worms were categorized as paralyzed, which did not move or just moved the head when gently touched with a platinum loop. Paralysis was scored at 2-hour intervals until all worms were paralyzed. The assay was performed for three biological replicates with 60 worms per trial.

#### 2.6.2. A*β*-Induced Chemotactic Dysfunction Assay

A*β*-induced chemotaxis assay was performed as previously described [21]. The eggs of CL2355 or CL2122 (a control strain) transgenic strains were treated with or without HT at 16°C for 36 h and then at 25°C for 36 h.Approximately 60 worms were placed in the center of a chemotaxis agar plate (9 cm). Before the placement, 1 *μ*l of 0.25 M sodium azide along with 1 *μ*l of odorant (0.1% benzaldehyde in 100% ethanol) was added to one side of the plate (“attractant” spot). On the opposite spot, 1 *μ*l of control odorant (100% ethanol) and 1 *μ*l of 0.25 M sodium azide were added. The plates were incubated for 1 h at 25°C, and then, the number of worms in each quadrant (up to 2 cm radius from the spots) was scored. The chemotaxis index (CI) was calculated (CI = (number of worms in attractant quadrants – number of worms in control quadrants) / total number of scored worms).

### 2.7. polyQ-Dependent Paralysis Assay

Cultured as described in the lifespan assay, AM140 transgenic worms cultured with or without HT were defined as paralyzed if they failed to move forward upon the tail prodding as previously described [22]. The assay was performed for three independent trials with 60 worms per trial.

### 2.8. Age Pigment Fluorescence Assay

To assess the accumulation of age pigment, age-synchronized worms (N2 strain) were treated with or without HT for 7, 12, and 17 days. Subsequently, the worms were anesthetized and mounted on a glass slide. Animals were selected randomly to submit to fluorescence microscopy with a GFP filter (Axio Imager Z2, Carl Zeiss AG, Jena, Germany). Considering that it is easy to confuse the strong near-death green autofluorescence with the increases in GFP in worms by green autofluorescence (through the GFP filter set) [23], the nematode individuals selected had strong vitality and were able to respond quickly to stimulation. Images were taken from at least 15 worms per group with a 10x objective. ImageJ software (NIH Image) was used to analyze the average pixel intensity of each worm in four independent trials.

### 2.9. Intestinal Fat Deposition Assay

As previously established, Oil Red O staining was used to determine fat content, but the freeze-thaw steps were omitted [24]. Briefly, worms were fixed in 4% paraformaldehyde, dehydrated in 60% isopropanol, and then stained with Oil Red O staining solution for 12 hours in the black with gentle rocking. Deposits of intestinal fat were imaged 40 times with an SLR camera (Canon Eos 5D Mark II) and quantified using ImageJ software (NIH Image). The assay was performed in three independent trials with at least 45 wild-type N2 worms.

### 2.10. Locomotion Assay

Locomotion assay was performed in three different life stages (the early, middle, and mid-late) as described [25]. Worms were observed and classified (classes A, B, and C) by a stereomicroscope according to the response of worms to stimuli. The movement behavior of 60 worms was detected after a one-minute recovery period. Three independent experiments were performed.

### 2.11. Pharyngeal Pumping Assay

The pharyngeal pumping frequency was measured by monitoring the movement of the pharynx terminal bulb under a stereomicroscope for 30 s (15 worms). Three independent experiments were performed in duplicate.

### 2.12. E. coli OP50 Bacterial Growth Assay

The bacterial growth assay of *E. coli* OP50 was performed as described previously [24]. The determination was set at OD600 for 12 h (*n* = 3).

### 2.13. Sample Characterization by Liquid Chromatography Coupled with Quadrupole Time of Flight Mass Spectrometry (LC-Q-TOF-MS/MS) Analysis

LC-Q-TOF-MS/MS analysis was used to investigate the sample components. The chromatographic system consisted of an Eclipse Plus C18 (2.0 × 100 mm, 1.8 *μ*m, Agilent) with a binary pump, degasser, column oven, and autosampler performed on a Uplc1290-6540B Q-TOF (Agilent Technologies, Palo Alto, CA, USA). Separation of HT was achieved on a 30 min linear gradient of acetonitrile (A) and 0.2% formic acid in ultrapure water (B), increasing from 5 to 95% B, with a 2 min hold at 95% B and a 4 min postrun at 10% B. The injection volume was 1 *μ*l, and the flow rate was 0.4 ml/min at 40°C. For the online TOF-MS and TOF-MS/MS analysis, experimental operation parameters were set as follows: nozzle voltage, 500 V; skimmer, 65 V; gas flow, 8 l/min; gas temp, 300°C; and nebulizer, 45 psi. The collision energy was set at 0 eV for negative and positive ion mode. The mass scan was over the range of *m*/*z* 105-1700 for both modes. Data analysis was carried out using the Agilent Mass Hunter Qualitative software (version B.07.00). Accurate mass scan data were mined using the find by molecular feature, find by formula, and find by targeted MS/MS and molecular formula generator algorithms.

### 2.14. SOD-3::GFP Visualization and Quantification Assay

To assess SOD-3::GFP expression, CF1553 worms were treated with or without HT for 96 hours as previously described [25]. The fluorescence of SOD-3::GFP was imaged using a fluorescence microscope with suitable filter and a 10x objective (Axio Imager Z2, Carl Zeiss AG, Jena, Germany) and analyzed using ImageJ software (NIH Image) in the whole body region (>15 worms/trial, with a total of 3 trials).

### 2.15. DAF-16::GFP Visualization and Delocalization Assay

To determine the subcellular localization of DAF-16::GFP, TJ356 worms were treated with or without HT for 96 hours as previously described [26]. The fluorescence of DAF-16::GFP was imaged using a Leica TCS SP8 (Buffalo Grove, IL) laser scanning confocal microscope with 100-fold magnification (excitation wavelength: 488 nm; emission wavelength: 510–550 nm). At 30 worms per treatment were scored as cytosolic, intermediate, and nuclear. The number of worms in each class was calculated from at least three biological replicates.

### 2.16. Statistical Analysis

It was considered to be a statistically significant difference if *p* < 0.05 applying one-way ANOVA followed by post hoc comparison (Tukey-Kramer test) for multiple comparison and independent samples *t*-test for two group comparisons (SPSS software, version 16). Survival curves were analyzed using Mantel-Cox log-rank test by using GraphPad Prism (GraphPad Prism, version 5.00 for Windows). The two-stage procedure was used when there was an intersection in the survival curve [27]. All data are expressed as mean ± SD, calculated in three independent experiments. Different letters in columns indicate that the values are significantly different (*р* < 0.05). In the survival curve, ^∗^*p* < 0.05, ^∗∗^*p* < 0.01, and ^∗∗∗^*p* < 0.001 compared with the control check (CK) group.

## 3. Results and Discussion

### 3.1. Effects of HT on Lifespan of C. elegans

**Figure 1:**
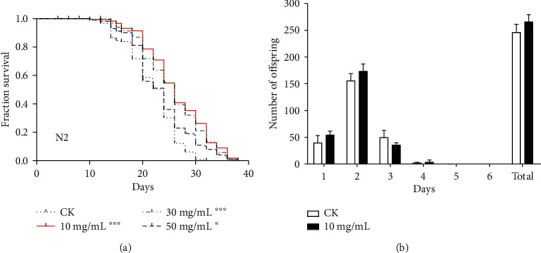
Effects of HT on lifespan and fertility of *C. elegans*: (a) lifespan curves of HT on N2 worms (^∗^*p* < 0.05, ^∗∗^*p* < 0.01, and ^∗∗∗^*p* < 0.001 compared with the CK group, the log-rank test); (b) the effect of HT on worm fecundity at the optimum longevity dose. The results are presented as the mean ± SD.

First, we studied the effect of HT on the lifespan of *C. elegans*. Interestingly, the lifespan curves of the three test concentrations (10 mg/ml, 30 mg/ml, and 50 mg/ml) all showed a significant right shift compared to those of the control group (*p* < 0.0001 by the log-rank test for 10 mg/ml and 30 mg/ml, *p* = 0.0260 by the log-rank test for 50 mg/ml), suggesting that the HT sample had the benefit of extending lifespan in *C. elegans* (Figure 1(a)). Moreover, the most significant longevity effect of HT was found at the concentration of 10 mg/ml, which increased the mean lifespan by 20% (26.09 days vs. 21.70 days) and extended the maximum lifespan by 7.50 days (37.00 days vs. 29.50 days) (Table 1). It was evident that HT had the potential to prolong the lifespan of *C. elegans*.

Since the optimal lifespan extension effect of HT was observed at the dose of 10 mg/ml, we further used the concentration to investigate and test whether HT had an adverse effect on the number of progenies. As shown in Figure 1(b), there was no significant difference in the daily number of offspring and total number of offspring in worms treated with control or HT, indicating that HT neither delayed nor inhibited fertility. So HT did not produce obvious reprotoxicity to worms and the beneficial effects of HT on lifespan might be independent of reproductive capacity in *C. elegans* at the dose of 10 mg/ml.

### 3.2. Effects of HT on Oxidative, Heat, and Proteotoxic Stresses in C. elegans

**Figure 2:**
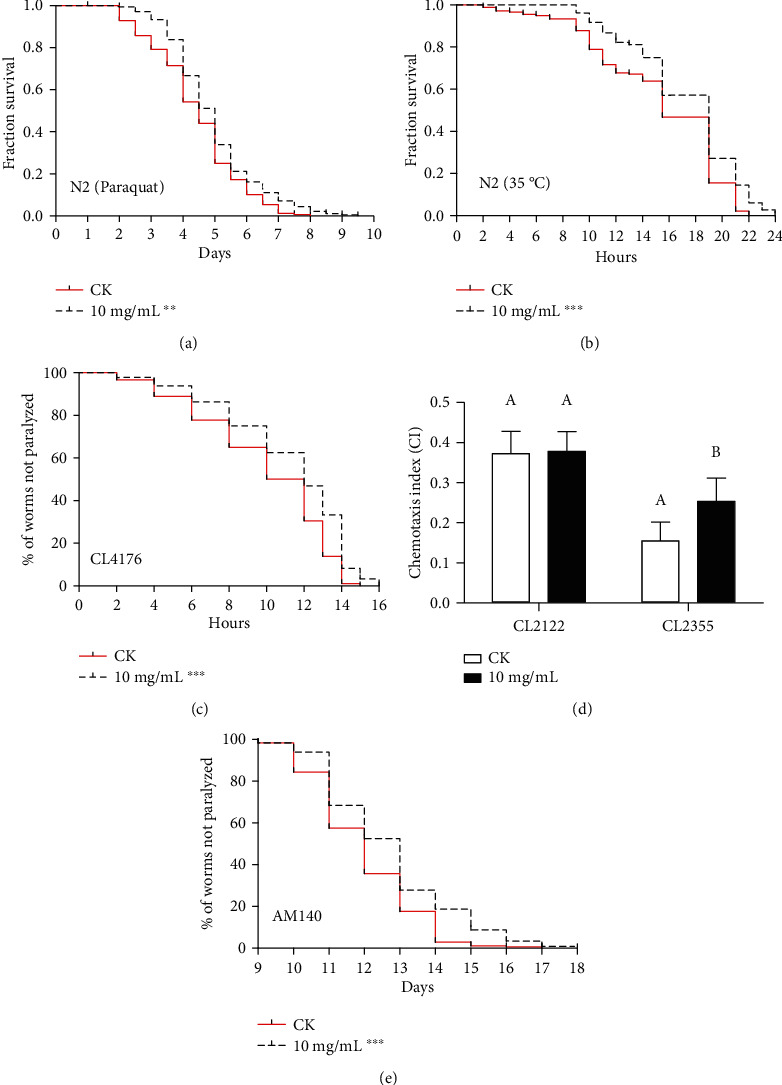
Effects of HT on oxidative, heat, and proteotoxic stresses in *C. elegans*: (a) survival curve of worms exposed to paraquat-induced oxidative stress after incubation with HT; (b) survival curve of worms exposed to35°C for heat stress testing after the nematodes were pretreated with HT at 20°C for 96 hours; (c) paralysis curve of CL4176 worms; (d) the A*β*-induced chemotactic dysfunction index after treatment of HT; (e) paralysis curve of AM140 worms. All data are presented as the mean ± SD of at least three independent experiments. Different letters above bars indicate a significant difference at *p* < 0.05 and ^∗∗^*p* < 0.01 and ^∗∗∗^*p* < 0.001 compared with the CK group in the survival curve.

A large body of credible evidence suggested that longevity was often accompanied by increased resistance to environmental stress, and stress resistance might be a determining factor in the lifespan of *C. elegans* [28]. Firstly, we investigated two stress conditions to comprehensively evaluate stress tolerance, which were paraquat-induced oxidative stress and heat stress. Considering that a large number of the matricidal death (“bagging”) of nematodes will happen if the nematodes are exposed to paraquat at the reproductive period [29], oxidative stress was carried out after the reproductive period (the 10th day) to avoid the matricidal death. Heat stress was measured on the 4th day of the life cycle, which is the peak period of adult worm vitality. The survival curve of HT treatment was different from that of the control group (*p* = 0.0034 and *p* < 0.0001 by the log-rank test for oxidative stress and heat stress, respectively, Figures 2(a) and 2(b)). In oxidative stress and heat stress protection assay, HT treatment significantly increased the mean lifespan (prolongation of 12 and 15%, respectively, Table 1) and the maximum lifespan (extended from 21.67 to 23.35 days and from 7.50 days to 9.00 days, respectively, Table 1). It suggested that HT had the potential to protect against stress.

It is increasingly clear that protein misfolding, which is caused by cellular stress, leads to cell dysfunction [30]. When these events occur in neurons, they may lead to neurodegenerative diseases over time [30]. Secondly, to determine the effect of HT on proteotoxic stress, we applied transgenic *C. elegans* CL4176, which expresses an aggregating amyloid-beta (3-42) peptide (A*β*(3–42)) in muscle tissue [31]. In humans, A*β*(3-42) aggregation is considered to be a key pathological factor in Alzheimer's disease [32]. We found that the HT treatment group exhibited a significantly right-shifted paralysis curve in CL4176 (*p* < 0.0001 by the log-rank test, Figure 2(c)), suggesting a significant delay in the paralysis time overall. HT treatment significantly delayed the mean paralysis time by 16%, with the maximum paralysis time from 14.33 to 15.44 hours in CL4176 (Table 1). Subsequently, we studied the effect of HT on A*β*-induced neurotoxicity using CL2355 strain expressing human A*β*_1-42_ in neuronal cells (a phenomenon leading to chemotactic dysfunction). As shown in Figure 2(d), the CI of the blank strain CL2122 (no A*β* expression) was lower than that of the untreated A*β* strain CL2355. These data demonstrated that CL2355 nematodes suffered more damage in neurons sensitive to the attractant benzaldehyde. Strikingly, the CI of the CL2355 strain treated with HT was significantly higher than that of the untreated CL2355. These results indicated that HT had a neuroprotective effect against A*β*-induced paralysis in *C. elegans*. Furthermore, another transgenic nematode model AM140 was applied. The AM140 worm expresses polyglutamine (polyQ) protein in body wall muscle cells, which is characteristic of several neurological diseases [32]. A similar inhibitory paralysis effect was also observed in AM140 (Figure 2(e)). In polyQ-dependent paralysis, not only a significantly different paralysis curve was exhibited in the HT-treated AM140 worms, but also the mean paralysis time and maximum paralysis time were significantly prolonged (Figure 2(e) and Table 1). Collectively, HT conferred stress resistance against different stressors, including oxidative stress, heat stress, and proteotoxic stress, which might alleviate protein misfolding and maintain protein homeostasis.

### 3.3. Effects of HT on the Reduction of Fat Accumulation and Age Pigment in C. elegans

**Figure 3:**
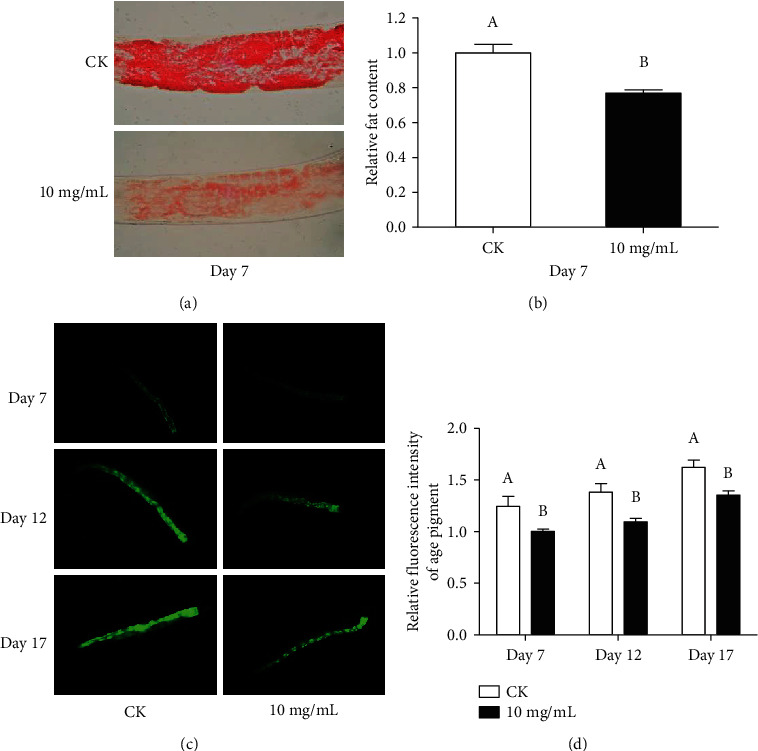
Effects of HT on the accumulation of fat and age pigments in *C. elegans*: (a) representative Oil Red O staining of nematode intestinal fat for control group and HT group; (b) the relative intensity of Oil Red O staining in the HT group and the control group; (c) the typical age pigment fluorescence images of HT group and control group in the early, middle, and mid-late stages (days 7, 12, and 17, respectively); (d) the effect of HT on the age pigment of N2. Bars represent mean ± SD of at least three experiments, and different letters above bars indicate a significant difference at *p* < 0.05.

The prevalence of obesity has risen sharply, adversely affecting longevity and health throughout the life cycle [33]. Since excessive fat accumulation has a serious impact on the quality of life, we investigated the effect of HT on the fat accumulation of *C. elegans*. The staining intensity of HT-treated nematodes was significantly weaker than that of the control group, as monitored by Oil Red O staining (Figure 3(a)). Using ImageJ quantitative analysis of the staining intensity, the result showed a 23% reduction in the HT treatment group (Figure 3(b)). It was suggested that HT had the potential to reduce fat accumulation in *C. elegans*.

A conservative feature of aging is the accumulation of age pigments with age [34]. These fluorescent compounds include autofluorescence lipofuscin and advanced glycation end products [34]. The accumulation of age pigments, the biomarkers of aging, and the healthspan of *C. elegans* were comprehensively evaluated in the different stages (days 7, 12, and 17 represented the early, middle, and mid-late stages, respectively). In wild-type animals, age pigments accumulated with age (Figures 3(c) and 3(d)). However, HT treatment significantly alleviated the deposition of age pigments by 19%, 21%, and 16% at different stages, respectively (Figures 3(c) and 3(d)).

### 3.4. Effects of HT on Behavioral Mobility in C. elegans

**Figure 4:**
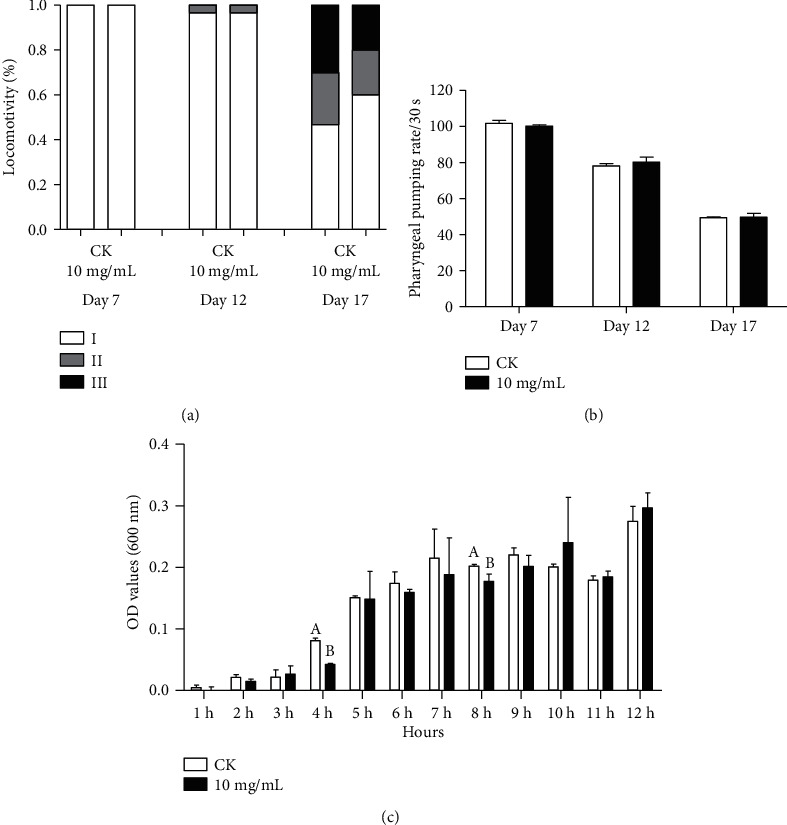
Effects of HT on behavioral mobility *C. elegans*: (a) the effect of HT on the age-related decline in exercise of N2 after 7, 12, and 17 days of treatment. Individuals were classified according to their locomotion: a free movement, B-stimulated motion, and C-stimulated weak motion; (b) the effect of HT on the frequency of pharyngeal pumping of N2; (c) the curve of *E. coli* OP50 bacterial growth after treatment of HT. All data are expressed as mean ± SD of at least three experiments.

We further investigated the effects on behavioral mobility. First, the locomotion was investigated according to three levels. And we focused on the locomotion in the early, middle, and mid-late stages. On day 17, compared to the control group, HT treatment group increased significantly the number of free-moving worms but decreased the number of individuals which could not complete the systemic movement after stimulation (Figure 4(a)). Second, the pharynx is the main organ of worm feeding, so its muscle contractility affects the absorption and metabolism of bacteria and HT [35]. We further investigated the frequency of pharyngeal pumping, a marker for food intake, in three different periods. Strikingly, no significant difference was observed between the treatment and the control groups (Figure 4(b)). Considering the antibacterial potential of HT, we speculated that the absence of improvement in the pharyngeal pumping frequency of worms was due to the growth inhibition of *E. coli* OP50, as a marker for food availability in *C. elegans*. However, this speculation was rejected since the result showed that growth of *E. coli* OP50 had not been affected after 9 hours (Figure 4(c)). Obviously, HT treatment could significantly improve the locomotion of nematodes but had no effect on feeding behavior as previously described by *Hibiscus sabdariffa* L. extract [36]. It also showed that the calorie restriction effect might not be the key reason for antiaging of HT.

### 3.5. Effect of HT on Delaying Aging Might Be Attributed to Its Components

To further analyze the composition of HT, LC-Q-TOF-MS/MS was performed. Representative total ion chromatograms (TIC) of HT are shown in Figure S1. Twenty compounds were tentatively identified in HT according to the previous reports (eighteen polyphenols and two terpenoids, Table 2) [5]. The main components were polyphenols, such as rosmarinic acid, isochlorogenic acid B, isochlorogenic acid C, glycyrrhizic acid, and protocatechualdehyde. In previous investigations [37], we found that rosmarinic acid improved antioxidant activity and healthspan in *C. elegans*. Isochlorogenic acids, as bioactive components of Lonicera japonica Thunb., has a wide range of biological activities, including antioxidant, antibacterial, and antiviral [38]. Strikingly, studies had shown that isochlorogenic acid B had a protective effect on liver fibrosis in mice with nonalcoholic steatohepatitis. And isochlorogenic acid C was found to prevent enterovirus 71 infection by regulating the redox homeostasis of glutathione [39]. Glycyrrhizic acid and protocatechualdehyde had been proven to have powerful antioxidant effects, which are considered to be promising potential human antiaging compounds [40, 41]. In general, natural antioxidants are considered an effective strategy for antiaging. Several studies demonstrated that supplementing with antioxidants from natural sources, such as plant extracts, could prolong the lifespan of many model organisms [42]; however, whether the effectiveness of antioxidants could be used as an indicator of longevity stills remain unclear. For example, Pun et al. pointed out that there was no intrinsic relationship between antioxidant activity and longevity of nematodes exposed to six plant extracts [43]. In this study, we also wondered whether the HT-mediated longevity was associated with increased antioxidant activity in nematodes. We found that HT enhanced the nematode resistance to paraquat-induced oxidative stress. So the effect of HT on delaying aging might be attributed to the excellent oxidative stress tolerance ability and health improvement potential of its components. But the topic of whether HT prolonged lifespan by improving antioxidant defense mechanisms deserved further discussion.

### 3.6. Effects of HT on Protein Expression of SOD-3::GFP in C. elegans

**Figure 5:**
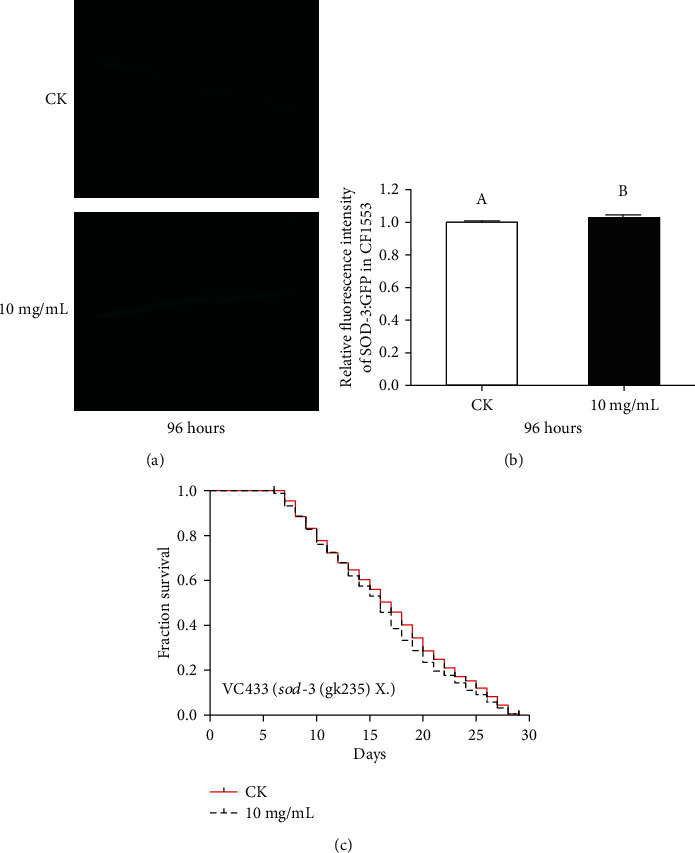
Effects of HT on the SOD-3 expression in *C. elegans*: (a) representative fluorescence images of SOD-3::GFP in CF1553 worms with or without HT treatment; (b) the effect of HT treatment on the expression of SOD-3::GFP in CF1553. Bars represent mean ± SD, and the different letters above bars indicate a significant difference at *p* < 0.05; (c) lifespan curves of HT on *sod-3* mutant worms.

SOD-3, a mitochondrial Fe/Mn superoxide dismutase, uses ROS as a substrate to convert substances that produce cell damage into safer compounds [44]. To explore the contribution of SOD-3 to the health benefits mediated by HT, we applied a SOD-3::GFP reporter strain CF1553 for visualization of SOD-3 expression. The HT-treated group showed a higher intensity of SOD-3::GFP expression than the control group (Figures 5(a) and 5(b)). To further verify whether gene *sod-3* was involved with HT-mediated longevity, we measured the lifespan of *sod-3* null mutant VC433. Consistent with this result, our results showed that the lifespan extension was completely abolished in the *sod-3* mutant strain, revealing a SOD-3-dependent mechanism in HT-mediated longevity (Figure 5(c)). Therefore, we speculated that the intracellular stress alleviation of HT was positively correlated with increased SOD-3 expression, which lowered the intrinsic pressure of the cells to maintain homeostasis [45].

### 3.7. Effects of HT on the Subcellular Localization of DAF-16 in C. elegans

**Figure 6:**
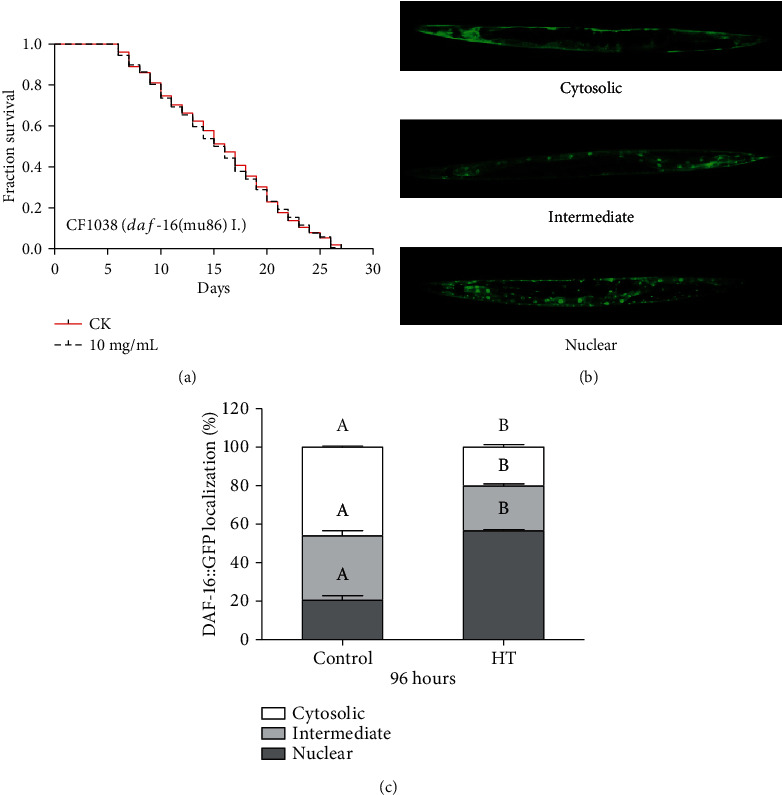
Effects of HT on the DAF-16 expression in *C. elegans*: (a) lifespan curves of HT on *daf-16* mutant worms; (b) three typical distributional fluorescence images of DAF-16 in the transgenic *C. elegans* strain TJ356, including cytosol localization, intermediate localization, and nuclear localization; (c) the effect of HT treatment on the subcellular distribution of DAF-16 in TJ356. Bars represent mean ± SD, and the different letters above bars indicate a significant difference at *p* < 0.05.

In *C. elegans*, the transcription factor DAF-16, the ortholog of mammalian FoxO3a (also known as FKHR-L1), is a key regulator of lifespan as well as resistance to oxidative or heat stress in response to insulin/insulin-like growth factor signaling (IIS) pathway [46]. And *sod-3* is a direct downstream gene of *daf-16* [44]. To investigate whether *daf-16* could play a role in lifespan extension by HT, we investigated the lifespan of HT acting on *daf-16* mutants (GR1307). HT failed to extend the lifespan of *daf-16* deletion mutants, indicating that DAF-16 might be a key longevity factor in the HT-dependent longevity mechanism (Figure 6(a)). Considering that the nuclear localization of DAF-16 is the essential prerequisite for DAF-16 transcriptional activation [47], we examined the effect of HT on the nuclear localization of DAF-16 by using DAF-16::GFP transgenic worms TJ356. It was found that HT affected the subcellular distribution of DAF-16 and induced translocation of DAF-16 from the cytoplasm to the nucleus (Figures 6(b) and 6(c)). Specifically, HT treatment induced an increase in nuclear and intermediate DAF-16::GFP translocation, especially in nuclear localization from 20% to 57% (Figures 6(b) and 6(c)). Meanwhile, the cytosolic DAF-16::GFP ratio was decreased from 46% to 20% (Figures 6(b) and 6(c)). Therefore, these results collectively suggested that HT-mediated improvement of lifespan and healthspan might be positively linked to the IIS pathway, which might activate the target gene *sod-3* by regulating the nuclear localization of DAF-16 in *C. elegans*.

## 4. Conclusion

In this study, we found that 10 mg/ml HT did not produce obvious toxicity to worms, which prolonged lifespan and improved the health of *C. elegans* without significant damage to physiological function. First, the lifespan was improved by HT without affecting fertility. Meanwhile, the stress resistance and proteotoxicity-induced paralysis were significantly improved. Moreover, HT significantly inhibited the accumulation of lipid and age pigmentation. Finally, we initially elucidated that the HT-mediated health-enhancing effect was positively associated with increased SOD-3 expression and enhanced nuclear translocation of DAF-16 in an IIS-dependent manner. In conclusion, this work provided the first insight into the health promotion role of HT and elucidates its underlying mechanism. Further studies on dietary HT intake in mouse models or humans are worthy of future research.

## Figures and Tables

**Table 1:** Statistical analysis of survival time of *C. elegans*.

Genotype	Treatment (mg/ml)	Mean time^(1)^	Maximum time^(2)^	% effect^(3)^	*p* value^(4)^
N2	0	21.70 ± 1.86^a^	29.50 ± 1.91^a^	/	/
	10	26.09 ± 0.53^b^	37.00 ± 1.15^b^	20	<0.0001
	30	25.28 ± 2.41^ab^	35.50 ± 2.52^b^	16	<0.0001
	50	23.39 ± 1.43^ab^	34.50 ± 3.42^ab^	8	0.0260
	0 (paraquat)	4.43 ± 0.26^a^	7.50 ± 0.50^a^	12	0.0034
	10 (paraquat)	4.95 ± 0.09^b^	9.00 ± 0.50^b^		
	0 (heat)	15.36 ± 0.28^a^	21.67 ± 0.58^a^	15	<0.0001
	10 (heat)	17.28 ± 0.84^b^	23.33 ± 0.58^b^		
CL4176	0	10.02 ± 0.69^a^	14.33 ± 0.58^a^	16	<0.0001
	10	11.67 ± 0.34^b^	15.33 ± 0.58^b^		
AM140	0	11.95 ± 0.22^a^	15.67 ± 1.53^a^	9	<0.0001
	10	13.08 ± 0.29^b^	17.67 ± 0.58^b^		
VC433	0	17.57 ± 0.22	28.33 ± 0.58	/	/
	10	17.08 ± 0.25	28.00 ± 1.00		
CF1038	0	16.65 ± 0.12	26.33 ± 1.15	/	/
	10	16.34 ± 0.32	26.33 ± 0.58		

^(1)^Mean time = (1/*n*)∑_*j*_((*x*_*j*_ + *x*_*j*+1_)*x*_*j*_ + *x*_*j*+1_/2)*dj*, where *j* is the age category, *d*_*j*_ is the number of worms that died in the age interval (*x*_*j*_, *x*_*j*+1_), and *n* is the total number of worms. ^(2)^The maximum time is the time at which fraction survival equals 0%. ^(3)^% effect was calculated by (*T* − *C*)/*C*∗100, where *T* is the mean time of worms treated with HT and *C* is the mean time of control. ^(4)^*p* value was calculated using the log-rank test by comparing the treated group with control.

**Table 2:** Tentative identification of chemical constituents of HT by UPLC/Q-TOF-MS/MS.

RT (min)	Mass	Abund	Percentage composition	Formula	Diff (ppm)	Significant IonMz	Tentative identification
7.045	516.1273	354272	6.00944	C_25_ H_24_ O_12_	1.11	515.1202	Isomeric di-O-caffeoylquinic acid
7.625	404.1326	150559	2.5539	C_17_ H_24_ O_11_	1.72	403.1253	Secoxyloganin
8.569	610.1543	160513	2.722748	C_27_ H_30_ O_16_	1.56	609.1472	Rutin
8.793	464.0954	150392	2.551067	C_21_ H_20_ O_12_	-0.14	465.1029	Isoquercitrin
8.901	448.1006	186538	3.164204	C_21_ H_20_ O_11_	0.01	449.1079	Trifolin
8.909	462.0797	102139	1.732562	C_21_ H_18_ O_12_	-0.21	463.0872	Scutellarin
8.909	154.0266	102139	1.732562	C_7_ H_6_ O_4_	-0.1	463.0872	Protocatechuic acid
9.273	594.1594	247027	4.190266	C_27_ H_30_ O_15_	1.53	593.1523	1-Hydroxybenzotriazole
9.274	198.0528	159629	2.707753	C_9_ H_10_ O_5_	-0.02	595.1658	Syringic acid
9.298	516.1279	602707	10.22359	C_25_ H_24_ O_12_	2.13	515.1208	Isochlorogenic acid C
9.431	138.0317	446001	7.56542	C_7_ H_6_ O_3_	0.17	137.0244	Protocatechualdehyde
9.513	516.1276	243848	4.136341	C_25_ H_24_ O_12_	1.6	515.1204	Isochlorogenic acid A
9.803	718.1547	173276	2.939244	C_36_ H_30_ O_16_	1.81	717.1476	Salvianolic acid B
9.944	516.1277	666790	11.31062	C_25_ H_24_ O_12_	1.7	515.1206	Isochlorogenic acid B
10.11	360.0859	755269	12.81147	C_18_ H_16_ O_8_	3.75	719.1636	Rosmarinic acid
10.59	418.127	192063	3.257924	C_21_ H_22_ O_9_	1.38	417.1198	Liquiritin
10.773	446.0847	275568	4.674401	C_21_ H_18_ O_11_	-0.4	447.0921	Baicalin
10.797	718.1545	229366	3.890686	C_36_ H_30_ O_16_	1.56	717.1473	Salvianolic acid E
11.477	592.1801	148132	2.512731	C_28_ H_32_ O_14_	1.58	637.1786	Linarin
14.94	822.4041	549030	9.313078	C_42_ H_62_ O_16_	0.32	823.4114	Glycyrrhizic acid

## Data Availability

The data used to support the findings of this study are included within the article and the supplementary information file.

## Abbreviations

A*β*: Alzheimer *β*-amyloid
*C. elegans*: *Caenorhabditis elegans*
CI: Chemotaxis index
CK: Control check
*E. coli* OP50: *Escherichia coli* strain OP50
IIS: Insulin/insulin-like growth factor signaling
LC-Q-TOF-MS/MS: Liquid chromatography coupled with quadrupole time of flight mass spectrometry
NGM: Nematode growth medium
polyQ: Polyglutamine
TIC: Total ion chromatograms
HT: Herbal tea.

## Supplementary Materials

Supplementary materials Figure S1: representative TIC of HT. Table S1: analysis of various chemical constituents in HT.
