# Supplementary material for: Healthspan Improvements in Caenorhabditis elegans with Traditional Chinese Herbal Tea
Source: Oxid Med Cell Longev. 2020 Dec 16;2020:4057841. doi: 10.1155/2020/4057841 (PMC7787765; doi:10.1155/2020/4057841)
Supplement: Supplementary materials — Figure S1: representative TIC of HT. Table S1: analysis of various chemical constituents in HT. [file 4057841.f1.docx]

**Supplementary Information**

**Healthspan improvements in *Caenorhabditis elegans* with traditional Chinese herbal tea**

## 1. Rrepresentative total ion chromatograms (TIC)

**Figure S1.** Rrepresentative TIC of HT. (A) TIC of HT in positive ion mode. (B) TIC of HT in negative ion mode.

**Fig. S1**

A


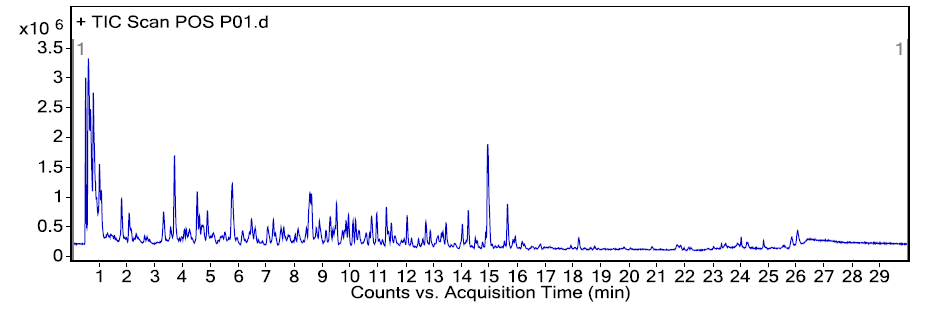


B


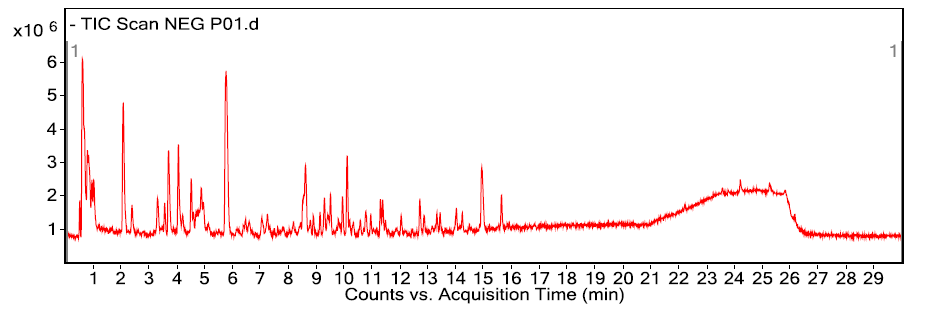


## 2. Analysis of various chemical constituents in HT

**Table S1 Analysis of various chemical constituents in HT**

| Assay | Content | Unit |
| --- | --- | --- |
| total organic acids | 3.17±0.26 | g/kg |
| total flavonoid content | 244.77±3.95 | g/kg Rutin equivalent |
| total saponin content | 60.86±2.61 | g/kg Ginsenoside Rg1 equivalent |
| water-soluble polysaccharide content | 100.41±7.84 | g/kg d-glucose equivalent |
